# The Silent Pandemic: The Psychological Burden on Frontline Healthcare Workers during COVID-19

**DOI:** 10.1155/2021/2906785

**Published:** 2021-09-30

**Authors:** Luise J. Froessl, Yazan Abdeen

**Affiliations:** Pulmonary and Sleep Physicians of Houston, P.A. 501 Orchard Street, Webster, TX 77589, USA

## Abstract

This narrative review explores the full scope of harmful psychological effects of the COVID-19 (Coronavirus Disease of 2019) pandemic on FLHCWs (Frontline healthcare workers). Additionally, we highlight the risk factors for worse outcomes. A literature review identified 24 relevant papers included in this synthesis. The majority of studies reported a high number of mental health conditions in HCWs (Healthcare workers) overall. Working in the frontline setting was repeatedly identified as an independent risk factor for poorer mental health. Additional risk factors, such as gender, occupational pressure, and low level of support from hospital administration, family, and the community, were also commonly identified. In the past, defined interventions have been shown to mitigate the psychological impact of high-stress situations on frontline workers. This review is aimed at identifying individuals at higher risk to help effectively target preventative measures in future stress situations in our healthcare system.

## 1. Introduction

On March 11^th^, 2020, the WHO (World Health Organization) officially declared the COVID-19 outbreak a global pandemic, a mere four months after identifying the first cases of SARS-CoV-2 (Severe acute respiratory syndrome coronavirus 2) in Wuhan, China. The pandemic has had severe, wide-reaching effects on the functioning of societies, economies, and healthcare systems globally [1]. In addition to the ongoing health crisis, a correlated mental health crisis has been unfolding [2]. An online survey of the U.S. general population conducted in March 2020 found that almost half of the participants met the criteria for anxiety or depression, compared to a baseline rate of these conditions of about 19% and 24%, respectively [3]. The purpose of this narrative review is to summarize the deleterious psychological effects of the COVID-19 pandemic on FLHCWs (Frontline healthcare workers) as reported by existing literature. The ultimate goal is to facilitate the development and implementation of preventative measures for the future.

Medical and paramedical staff who work directly with COVID-19-infected patients make up FLHCWs. These individuals are a crucial element in the medical response to a global pandemic and are simultaneously uniquely vulnerable to the psychological stresses imposed by the pandemic. They have been faced with novel challenges in the workplace, all while adjusting to the same changes in daily life as the general public.

The studies included in this review commonly evaluated the prevalence of the following mental health conditions. GAD (Generalized Anxiety Disorder) is defined by the DSM-V (Diagnostic and Statistical Manual of Mental Disorders) as the existence of excessive worry or apprehensive expectation about an event or activity that is difficult to control associated with defined physical symptoms [4]. A depressed mood, or anhedonia, in association with defined somatic or nonsomatic symptoms, characterizes major depressive disorder [5]. PTSD (Post-traumatic stress disorder) is defined as the development of negative emotional and behavioral symptoms in response to exposure to actual or threatened death, serious injury, or sexual violence [6]. Burnout is a work-related syndrome that Maslach et al. define by three dimensions: exhaustion, cynicism, and inefficacy [7]. Insomnia, a complaint of dissatisfaction with sleep quantity or quality, and its relationship to the previously mentioned conditions were also frequently evaluated [8].

Adverse psychological impacts on FLHCW have previously been reported during emergent viral epidemics such as the SARS-CoV-1 (Severe acute respiratory syndrome coronavirus 1), MERS (Middle East Respiratory Syndrome), or swine flu outbreaks [9, 10]. McAlonan et al. additionally reported that stress levels were higher in SARS FLHCWs one year after the initial exposure [11].

## 2. Methods

The PRISMA (Preferred reporting items for systematic reviews and meta-analyses) methodology checklist was used as a guideline to adequately identify and select the relevant literature for this narrative review. Searches of PubMed and Google Scholar were conducted from December 20^th^, 2020, to January 27^th^, 2021. The following keywords were entered in various combinations: “COVID-19,” “pandemic,” “coronavirus,” “healthcare worker,” “frontline,” “psychiatric,” “psychological,” “mental,” “stress,” and “mental health.” The bibliographies of relevant papers were also reviewed manually to extract additional literature of interest. Of the results, 24 studies were included in this review. Only studies that were available in the English language and published in peer-reviewed journals were included. Studies with a sample size of fewer than 100 participants or review papers, meta-analyses, editorials, and commentaries were not considered for inclusion.

## 3. Results

A review of the literature resulted in the identification of 24 relevant papers examining the psychological effects of the pandemic on FLHCWs (Table 1). Twenty-three of these papers used a cross-sectional, observational design and were conducted using an online survey completed by voluntary participants [12–34]. The remaining study was a matched cross-sectional study [35]. The number of participants included in each study ranged from 126 to 2285. The studies identified for this review originate from 10 different countries. Four studies included exclusively frontline workers [16, 26, 28, 33]; the rest consisted of healthcare workers with a subgroup of frontline workers. All but one study employed objective and scientifically verified scales to measure the presence of anxiety, depression, insomnia, and stress symptoms in included participants. Some also used additional questions or scales to evaluate perceived stress, PTSD, or other psychological factors.

All included studies found that adverse psychological effects were highly prevalent in healthcare workers. In most studies, direct contact with confirmed or suspected COVID-19 patients was an independent risk factor for adverse mental health outcomes. However, four of the 24 included papers showed no significant difference or a lower prevalence of mental health issues in the FLHCW group [15, 24, 27, 30].

The GAD-7 (Generalized Anxiety Disorder 7) and the PHQ-9 (Patient Health Questionnaire 9) were the most commonly employed scales used to evaluate participants for anxiety and depression, respectively. However, other scales such as the HADS (Hospital Anxiety and Depression Scale) or the DASS-21 (Depression, Anxiety, and Stress Scale 21) were also used. In the studies evaluated in this review, the anxiety was present in 15-50% of healthcare workers. The majority, but not all surveys, detected higher levels of anxiety in FLHCWs vs. non-FLHCWs. Cag et al. conducted a large-scale international study in March 2020 and found that 2/3 of FLHCWs screened positive for anxiety, with 17% showing severe anxiety levels [29]. Depression or depressive symptoms were detected in 15-65% of individuals depending on the study. Overall, FLHCWs were found to have significantly higher levels of anxiety and depression than HCWs. Significantly, one paper reported the significant association of depression with lower quality of life in the frontline nurses [16]. Additional risk factors were also examined. Being of female gender or being of younger age is an additional risk factor identified by multiple studies [18, 20, 29, 30, 34]. A history of chronic illness or having a mental disorder predisposed the individual to poor mental health [20]. Having experienced patient death from COVID-19 or the death of a close family member or friend from COVID-19 increased risk as well [20, 34].

Several studies reported posttraumatic symptoms and a perceived need for psychological support in healthcare workers. The risk for these conditions was independently associated with participating in frontline work [34].

Studies reported high levels of burnout in healthcare workers in Spain, Italy, Belgium, and China [19, 30, 31, 33].

The prevalence of insomnia in FLHCWs was also evaluated by several studies [14, 15, 17, 26, 28]. Three validated questionnaires were used to assess participants for insomnia: the ISI (Insomnia Severity Index), the PSQI (Pittsburgh Sleep Quality Index), and the AIS (Athens Insomnia Scale). Insomnia was common, with a prevalence of 35-75%. As reported with anxiety, depression, and other mental health symptoms, insomnia was also significantly more prevalent in medical professionals working directly with COVID-19 patients [35].

## 4. Discussion

### 4.1. Challenging Conditions for FLHCWs

Besides being exposed to the same mental stressors and societal shifts as the public, FLHCWs have faced many additional challenges due to the pandemic. Secondary to the nature of their work, they are burdened with a greater personal risk of exposure and of exposing family members. In addition, they face higher workloads, moral dilemmas, and the challenges that come with caring for acutely ill patients in their workplace [36].

A prospective, observational cohort study conducted among the American and British general public in March 2020 found that FLHCW had a significantly increased risk of testing positive for COVID-19 [37]. In the early stages of the COVID-19 pandemic, little was known about the risk of SARS-CoV-2 infection. One month after the initial cluster of cases was reported in Wuhan, China, the WHO first confirmed human-to-human transmission of the virus. Healthcare workers tasked with evaluating and treating suspected or confirmed cases were intensely exposed to this novel disease, even before the optimization of transmission prevention. Nguyen et al. found a significant correlation between PPE (Personal Protective Equipment) adequacy and the risk of testing positive for COVID-19. The supply of PPE was severely disrupted, especially in the early stages of the pandemic [38]. This shortage was reflected on a global scale [39].

At the pandemic's height, the shortage of resources, such as ventilators and ICU beds, made the consideration of rationing a necessity. *Rationing* is defined as the allocation of limited resources. In healthcare, this ultimately results in withholding of treatment from patients that may have potentially benefited. A substantial ethical dilemma exists for the individuals charged with making this decision [40].

The absence of an effective treatment for the COVID-19 illness increases the emotional burden of caring for infected patients. Physicians treating COVID-19 patients have had to cope with the difficulty of caring for often severely ill individuals with few options to propose. In addition, they have had to keep up with emerging knowledge and information and follow the rapidly changing recommendations and institutional policies [26].

FLHCWs have also been faced with marginalization in the community. HCWs have previously reported societal stigmatization and avoidance due to working directly with infected individuals during the SARS epidemic [41]. One study found that FLHCWs feeling higher levels of negative stigma in the community were more likely to report PTSD and alcohol use disorder symptoms [23].

A survey conducted in April 2021 by the Washington Post among FLHCWs evaluated the current subjective state of this population [42]. Not surprisingly, a high mental health burden was also found. However, 76% of the 1327 HCWs interviewed also reported feeling hopeful about the future. Majorities reported feeling optimistic and motivated when going to work. These findings are encouraging and paint a positive picture of the future.

### 4.2. Psychological Conditions in HCWs

Healthcare professionals are affected by high levels of stress, burnout, and other mental health conditions compared to other professions [43]. During a global health crisis, as working conditions are increasingly challenging, it is predictable that healthcare professionals will experience an increase in psychological stressors.

The relationship between stress and mental health conditions has been well studied. Acute stress responses can be adaptive and appropriate in certain situations. However, negative effects can ensue when the exposure to a stressor is prolonged or particularly intense. Evidence supports the existence of a causal link between depression and negative life events [44, 45]. Similar evidence exists for other mental health conditions such as GAD. A study on individuals directly affected by hurricanes showed a measurable biological and psychological impact with an increased risk of GAD [46]. An important element to note is that the response to stress can vary widely between different individuals exposed to the same stressor. It is therefore essential to identify the factors that favor poorer outcomes in order to effectively target preventative measures.

Several interacting factors contribute to determining what psychological effects a challenging situation will have on an individual. These factors are commonly divided into four distinct dimensions: individual (gender, age, previous mental health, and others), interpersonal (social support), institutional (workplace conditions), and community factors (stigma or support). It is essential to identify the adverse psychological effects on HCWs in intense stress situations such as the current pandemic (Table 2).

Although the extent of the COVID-19 pandemic has been much more significant than previous viral pandemics of this century, looking at past events may provide valuable information for our present situation. Both during the 2002-2004 SARS-Cov-1 epidemic and the MERS-CoV outbreak first detected in 2012, healthcare workers' mental health was closely scrutinized. Similarly to the current situation, healthcare workers in contact with SARS-CoV-1 reported high levels of stress and mental health conditions [11]. A study conducted during a 2015 MERS outbreak showed higher levels of PTSD in FLHCWs as well as quarantined patients [47] As early as 2003, risk factors were identified and preventative measures were proposed to avert similar outcomes in the future [48]. Unfortunately, the current data shows that mental health in FLHCWs is as poor, if not worse, than in FLHCWs during the previous epidemics. It is therefore more urgent than ever to develop and implement effective strategies to prevent these outcomes in the future.

#### 4.2.1. Depression and Anxiety

Symptoms of anxiety and depression were evaluated by most studies included in this review. They are closely related, and risk factors frequently overlap.

Previous studies conducted outside of the current pandemic have shown high levels of anxiety and depression in healthcare workers. Depression or depressive symptoms were found among 20-40% of resident physicians in a meta-analysis conducted in 2015 [49]. Similarly, 18% of hospital-employed nurses screened positive for depression in a 2012 survey [50]. Anxiety is also highly prevalent in HCWs and particularly common in emergency medical personnel [51]. Notably, studies have found that screening positive for anxiety or depression is associated with a significantly higher incidence of adverse safety outcomes in the healthcare setting [52].

These findings are consistent with what was reported by the majority of studies included in this review. As previously mentioned, most studies found a higher prevalence of GAD and depressive symptoms in FLHCWs than other HCWs.

Other risk factors were expected, as they are known to predispose an individual to GAD or depression, even outside of working in healthcare or a pandemic environment. Being of the female gender, younger age, and having a chronic illness or previous mental health disorder are known risk factors for poor mental health [53].

Working conditions were also determined to play an important role. Almost all studies found a higher prevalence of anxiety and depression in healthcare workers involved in frontline work. The risk of anxiety and depression increased when caring for more COVID-19 patients and with higher amounts of weekly working hours. Participants were also more likely to report symptoms of depression and anxiety if they felt a lower level of support from peers and supervisors and if they reported feeling less competent in caring for COVID-19 patients [18]. Having insufficient PPE to protect oneself and a lack of resources to care for patients were identified as two significant risk factors for anxiety and depression [26, 29].

One study conducted in Malaysia that specifically compared anxiety levels in FLHCW vs. non-FLHCWs found that non-FLHCWs had lower anxiety levels. The authors present the theory that FLHCWs generally have easier access to updated first-hand medical information on the virus and the pandemic [27]. The results of other studies have supported this theory by showing that anxiety levels were lower in individuals that reported being well informed about the pandemic or in individuals with higher levels of education [29, 22].

Low levels of trust in the government response to the pandemic and increased media exposure were also associated with GAD [23]. These findings suggest that developing an effective national response to an infectious disease outbreak may reduce the psychological effects on FLHCWs.

A survey conducted among FLHCWs found that individuals with higher anxiety and depression scores generally adopted harmful coping mechanisms and had lower self-efficacy than the group without negative psychology [22]. Active coping mechanisms that prevent adverse psychological reactions can be taught and learned and may represent an essential element to incorporate in mitigating adverse mental health outcomes on FLHCWs in the future.

These findings suggest several measures that may effectively reduce adverse mental health outcomes for FLHCWs. Limited working hours and a reduced patient load should be implemented whenever possible. Increasing support from management tiers of the healthcare establishment and providing easy and direct access to updated information to employees may reduce anxiety and depression levels. Furthermore, providing adequate PPE is crucial to reduce anxiety and increase confidence in healthcare workers.

#### 4.2.2. Posttraumatic Stress Disorder (PTSD)

In the context of previous viral epidemics such as SARS, it has been shown that FLHCWs had a higher prevalence of and more severe PTSD symptoms than other healthcare professionals [9]. A study assessing the long-term psychological effects of frontline work during the SARS outbreak in Hong-Kong HCWs did not find a higher level of perceived stress in FLHCWs. However, when reassessed at one year, high-risk HCWs had a significantly higher prevalence of chronic stress, PTSD, and other adverse mental health outcomes [11].

A survey conducted in Italian HCWs by Trumello et al. during the COVID-19 outbreak assessed perceived stress levels using the same scale used in the prior survey conducted by McAlonan et al. during the SARS epidemic [12]. As previously mentioned, the SARS FLHCWs showed significantly higher PTSD levels at one-year follow-up than other HCWs. The results identified by Trumello et al., therefore, may suggest that the prevalence of PTSD symptoms may continue to increase even after the acute phase of the pandemic passes. This study also showed that FLHCWs were twice as likely to seek psychological support as non-FLHCWs, suggesting that preventative programs would be sought out and potentially beneficial for these individuals.

Witnessing patient death while working in the COVID-19 sector was identified as a factor that may explain the higher prevalence of PTSD symptoms in FLHCWs. A survey of HCWs in Israel found a fourfold increase in PTSS (Post-traumatic stress syndrome) in HCWs that witnessed patient death while caring for COVID-19 patients [24]. Most healthcare workers are exposed to the passing of patients as a part of their professional lives. However, COVID-19 is known to be fatal even in relatively young and healthy individuals. Especially in certain extremely hard-hit areas throughout the pandemic, the volume of sick patients and patient deaths reached unprecedented and previously unseen levels, even for experienced HCWs [54].

A survey conducted among Chinese healthcare professionals found that PTSS were positively correlated with passive coping mechanisms and negatively correlated with active coping mechanisms [21]. This finding is encouraging, as it suggests that promoting the development of coping mechanisms in FLHCWs may reduce the incidence of PTSD development.

#### 4.2.3. Burnout

It has been previously shown that medical professionals are at particularly high risk for burnout. The syndrome is associated with adverse personal effects such as substance abuse, relationship difficulties, and even suicide. Professional ramifications are also common, such as increased medical errors, lower patient satisfaction, and reduced quality of care [55]. A survey conducted in 2011 evaluated burnout and job satisfaction levels in U.S. physicians. The study found that physicians were more likely to have burnout symptoms (37.9% vs. 27.8%) than a representative sample of the adult U.S. working public. The prevalence was particularly elevated in physicians working in the first line of care, such as emergency medicine specialists or general practitioners [43].

According to a study by Macía-Rodríguez et al., 40% of Spanish internists working as part of the COVID-19 response screened positive for burnout. Several other studies reported similar levels [31, 30, 33]. Independent risk factors included working directly with COVID-19+ patients and overtime work without compensation, as well as the fear of infecting relatives [19]. These levels of burnout syndrome are a reason for concern as they are known to translate to poorer patient outcomes.

A prospective cohort study conducted in Scotland from March to June 2020 found a significantly increased risk of hospitalization for COVID-19 in household members of FLHCW [56]. Due to this risk, FLHCWs often chose to remain separated from their families and friends to reduce the risk of infection. Isolation from the social circle, which generally represents a source of comfort and support, significantly increases psychological pressure on these individuals. The risk of burnout in FLHCWs was significantly more elevated in individuals that chose to move out of their family homes while doing frontline work [31].

As previously mentioned, FLHCWs often experience stigmatization in the community due to their heightened exposure and risk for infection. One survey found that HCWs that reported social avoidance due to their profession were also more likely to report higher levels of burnout [31].

#### 4.2.4. Insomnia

The association between insomnia and poor mental and physical health has been extensively characterized in the past [57]. It is known that poor sleep quality and insomnia contribute to lower professional performance [58].

FLHCWs reported longer sleep latency, shorter sleep duration, lower sleep efficiency, and worse daytime function [14]. Female participants were more vulnerable to sleep disturbances. As for anxiety and depression, insomnia was more common in nurses and other nonphysician HCWs [15, 17]. This may stem from the fact that physicians generally tend to have access to more current and updated information on the pandemic and the pathogen in question. By the nature of their work, nurses may have the most intense and consistent exposure to infected patients. Nurses that reported fear of COVID-19 were more prone to experience insomnia. Not surprisingly, an increasing number of night shifts or longer working hours also contributed to a higher prevalence of insomnia [28]. Receiving negative feedback from family and friends about joining frontline work increased the risk for poor sleep quality [13]. These findings are particularly concerning when considering that there is some evidence that sleep deprivation may represent an independent risk factor for COVID-19 infection [59].

As insomnia is closely related to, and correlated with, other mental health conditions such as anxiety, depression, and PTSD, it is probable that when implementing strategies to reduce these psychological conditions in FLHCWs, the prevalence of insomnia will decrease in parallel.

### 4.3. Solutions/Future Perspectives

Review of the current literature identified several groups of FLHCWs that are particularly vulnerable to poor mental health due to pandemic-related challenges inside and outside the workplace. Intrinsic factors may predispose an individual to poor mental health. The female gender is a known risk factor for many mental health conditions [53]. However, in the context of this global pandemic, there may be additional contributing factors to consider. With lockdowns imposed by many governments, schooling duties, parenting, and household duties have shifted from teachers, sitters, and household aids back to parents. Women have disproportionately taken on these responsibilities, frequently in addition to full-time jobs [60]. Younger, less experienced individuals and those with preexisting mental health conditions were also shown to be part of higher-risk groups for poor mental health outcomes [34, 18]. Awareness of risk factors in specific individuals will permit targeting preventative strategies effectively.

Feeling insufficient support from superiors in the workplace or the healthcare institution was a commonly identified factor predisposing to all adverse psychosomatic outcomes in FLHCWs [23]. The CDC has provided recommendations for healthcare personnel to improve coping and improve resilience during the pandemic [61]. Maintaining adequate mental health in FLHCWs is essential for an effective response to a global infectious disease outbreak. These recommendations should therefore be integrated when revising or creating guidelines for future pandemic responses.

The overwhelming majority of the literature found a positive correlation between increased occupational pressure and poor mental health. Multiple studies emphasized the importance of improving workplace conditions for FLHCWs, such as reducing work hours, improving medical equipment supply, and reducing congestion in wards [25, 31, 29].

A study on Chinese healthcare workers was aimed at developing individual growth from posttraumatic stress by implementing a novel psychological intervention. *Posttraumatic growth* is defined as a positive psychological change developed in response to coping with challenging circumstances. The study showed that the intervention induced positive effects in HCWs. An increased effect was seen in women, nurses, and college graduates [62]. These findings suggest that the implementation of psychological interventions can limit negative outcomes in FLHCWs and may even achieve increased psychological resilience.

### 4.4. Limitations

Several limitations of this review must be noted. First, the literature review was conducted in the English language, and only papers available in English were included.

The large majority of studies included in this review were designed as voluntary online questionnaires. Therefore, a significant sampling bias cannot be ruled out when including exclusively volunteers. All studies included in this review reported a majority of female responders. As mentioned, the female gender is an independent risk factor for many mental health conditions. It is not unlikely that the prevalence of mental health conditions may be overestimated due to the sampling bias and may not be entirely representative of healthcare workers as a whole. All studies were cross-sectional in design. This design does not allow for the determination of the causal relationships that may exist. Overall, the results reported in this review were identified by several studies and were coherent and plausible when considering previous research, therefore increasing confidence in the results.

## 5. Conclusion

Worldwide, thousands of FLHCWs have died due to infection with COVID-19 [63]. Healthcare workers and their family members are significantly more likely to be infected and hospitalized with COVID-19 than members of the general public [37]. Novel and unprecedented challenges in the workplace and daily life have resulted in a significant and concerning psychological impact on our FLHCWs. Although no significant difference in mental health problems was reported before the pandemic, frontline medical staff tend to report higher levels of anxiety, depression, sleep problems, and lower quality of life since the outbreak. Psychosomatic issues are also more prevalent with increasing levels of exposure to COVID-19 patients [25]. Maintaining the mental and physical health of FLHCWs is crucial for an effective response to a global pandemic. Increasing awareness of the intrinsic and environmental risk factors predisposing healthcare workers to adverse mental health outcomes is crucial to target interventions that are aimed at managing these conditions in future situations of extraordinary strain on healthcare systems.

## Figures and Tables

**Table 1:** 

Reference	Location and date range	Study type	Sample size	Topic of study	Key outcomes
Conti et al. 2020 [34]	Italy March 30^th^ to May 5^th^ 2020	Online cross-sectional survey	933 HCWs	Mental health status and psychological care needs of HCWs during the COVID-19 pandemic	(i) Poorer mental health: female gender, age < 40 years, having experienced death of a patient (ii) Lesser but significant effect on mental health: working in a highly infected area, frontline work

Trumello et al. 2020 [12]	Italy April 11^th^ to April 16^th^ 2020	Online cross-sectional survey	627 HCWs	Psychological adjustment in HCW during the COVID-19 pandemic, the effect of frontline work	(i) Higher psychological impact in FLHCWs (ii) Demand for psychological support is double in FLHCWs vs. non-FLHCWs

Que et al. 2020 [13]	China February 16^th^ to February 23^rd^ 2020	Online cross-sectional survey	2285 HCWs	Risk factors for the development of psychological problems in different groups of HCWs during the COVID-19 pandemic	(i) Poorer mental health: frontline work, receiving negative information about the pandemic, receiving negative feedback from family or friends (ii) Higher risk in nurses, paramedical professionals, lower risk in medical residents

Qi et al. 2020 [14]	China February 2020	Online cross-sectional survey	1306 HCWs	Sleep disturbances and mental health in FLHCWs compared to non-FLHCWs during the COVID-19 pandemic	(i) Poorer mental health: FLHCWs, female gender (ii) Anxiety and depression associated with poor sleep quality

Jahrami et al. 2020 [15]	Bahrain April 2020	Online cross-sectional survey	257 HCWs	Sleep quality in FLHCWs as compared to non-FLHCWs during the COVID-19 pandemic	(i) High prevalence of poor sleep quality, no significant difference between FL and non-FLHCWs (ii) Sex and professional background are most predictive of poor sleep quality

An et al. 2020 [16]	China March 15^th^ to March 20^th^ 2020	Online cross-sectional survey	1103 ED nurses	Prevalence of depression and quality of life in ED nurses during the COVID-19 pandemic	(i) Frontline nurses: higher depression, lower quality of life

Cai et al. 2020 [35]	China February 11^th^ to February 26^th^ 2020	Online, case-control, matched	1173 FLHCW vs. 1173 non-FLHCW	Comparison of the pandemic's psychological impact on FLHCWs vs. non-FLHCW	(i) Poorer mental health and insomnia in FLHCWs (ii) No significant difference in help-seeking behavior

Zhang et al. 2020 [17]	China January 29^th^ to February 3^rd^ 2020	Online, cross-sectional survey	1563 HCW	Insomnia and related psychological and social factors in HCWs during the COVID-19 pandemic	(i) 1/3 medical workers included had insomnia (ii) Increased risk for insomnia: work in isolation wards, lower level of education, worry of being infected, uncertainty about effectiveness of infection control measures

Elbay et al. 2020 [18]	Turkey March 10^th^ to March 15^th^	Online, cross-sectional survey	442 physicians	Anxiety, depression, and stress in physicians during the COVID-19 pandemic	(i) DAS-21 was higher in FLHCWs (ii) Poorer mental health: female gender, young age, frontline work, less work experience

Macía-Rodríguez et al. 2021 [19]	Spain May 2020	Online, cross-sectional survey	1015 HCWs	Impact of the COVID-19 outbreak on mental health and burnout syndrome in internists	(i) Higher burnout levels in FLHCWs, insufficient PPE, worry about infecting family, OH consumption, increased responsibility, longer work hours, and no rest
Erquicia et al. 2020 [20]	Spain March to April 2020	In-person, cross-sectional survey	395 HCWs	Analysis of the emotional state of healthcare professionals during the COVID-19 pandemic	(i) High prevalence of anxiety, depression, acute stress symptoms (ii) Increased risk of psychological distress in females, FLHCWs, and paramedical staff; feeling of having insufficient PPE; having experienced the death of a close person from COVID-19

Si et al. 2020 [21]	China February 23^rd^ to March 5^th^ 2020	Online, cross-sectional survey	863 HCWs	Psychological impact of the COVID-19 pandemic on HCWs	(i) High level of PTS symptoms (ii) Females, nurses, and FLHCWs have higher risk (iii) Perceived threat and passive coping increase risk for PTS

Xia et al. 2021 [22]	China February 3^rd^ to March 30^th^ 2020	Online, cross-sectional survey	126 HCWs	Psychological impact and coping mechanisms in medical staff during the COVID-19 pandemic	(i) Doctors and nurses had lower anxiety levels than administrative personnel (ii) Lower depression if bachelor's degree or above (iii) Negative coping mechanisms significantly correlated with anxiety/depression

Hennein et al. 2021 [23]	USA May 2020	Online, cross-sectional survey	1092 HCWs	Socioecological predictors of mental health outcomes in HCWs during the COVID-19 pandemic	(i) Higher levels of PTSD: female gender, lower team cohesion, higher levels of social stigmatization, higher media exposure

Mosheva et al. 2021 [24]	Israel April 19^th^ to April 23^rd^ 2020	Online, cross-sectional survey	828 HCWs	Comparison of PTSS, depression, and anxiety in FLHCWs vs. non-FLHCWs during the COVID-19 pandemic	(i) Anxiety and depression similar in both FL- and non-FLHCWs (ii) FLHCWs: higher risk of witnessing patient death, associated with higher PTSS (iii) Increased anxiety and depression with increased worry about being infected, worry about infecting family, mental exhaustion

Yi et al. 2020 [25]	China April 26^th^ to May 9^th^ 2020	Online, cross-sectional survey	723 HCWs	Psychosomatic status in different groups of HCWs during the COVID-19 pandemic	(i) Poorer mental health with intensity of contact with COVID-19 patients, higher occupational pressure

Barua et al. 2020 [26]	Bangladesh April 1^st^ to May 30^th^ 2020	Online, cross-sectional survey	370 frontline doctors	Anxiety, depression, sleep disturbance, and fear of COVID-19 among frontline doctors	(i) Inadequate resources to provide for patients contribute most to poor mental health symptoms

Mohd Noor et al. 2021 [27]	Malaysia May to July 2020	Online cross-sectional survey, comparative	306 HCWs	Anxiety in FLHCWs vs. non-FLHCWs during the COVID-19 pandemic	(i) Higher levels of anxiety were reported in the group of non-FLHCWs

Shen et al. 2021 [28]	China March 3^rd^ to March 10^th^ 2020	Online cross-sectional survey	643 frontline nurses	Levels of anxiety among frontline nurses during the COVID-19 pandemic and its association with insomnia and perceived stress	(i) High levels of anxiety correlated with insomnia, perceived stress, night shifts, fear of COVID-19, having experienced previous pandemics

Cag et al. 2021 [29]	International (75 countries) March 18^th^ to April 1^st^ 2020	Online, cross-sectional survey	1416 HCWs	Anxiety levels in international HCWs during the COVID-19 pandemic	(i) Increased anxiety with inadequate resources, female gender, young age, insufficient knowledge of COVID-19, living in high-income countries
Tiete et al. 2020 [30]	Belgium April 17^th^ to May 25^th^ 2020	Online, cross-sectional survey	647 HCWs	Differences in mental health between FLHCWs and non-FLHCWs	(i) High prevalence of anxiety, depression, and insomnia, no significant difference between FL and non-FLHCWs (ii) Worse mental health outcomes: nurses, young age, working in isolation wards, increased workloads

Lasalvia et al. 2021 [31]	Italy April 21^st^ to May 6^th^ 2020	Online, cross-sectional survey	1961 HCWs	Burnout levels and risk factors in HCWs working during the COVID-19 pandemic	(i) High risk of burnout: junior doctors working in FL settings, interpersonal avoidance, conflicts in the workplace, increased workload

Lai et al. 2020 [32]	China January 29^th^ to February 3^rd^ 2020	Online, cross-sectional survey	1257 HCWs	Mental health outcomes and associated factors in HCWs during the COVID-19 pandemic	(i) Poorer mental health outcomes: nurses, women, and FLHCWs

Hu et al. 2020 [33]	China February 13^th^ to February 24^th^ 2020	Online, cross-sectional survey	2014 frontline nurses	Mental health outcomes and associated factors in frontline nurses caring for COVID-19 patients	(i) Lack of social support is correlated with negative mental health outcomes (ii) Willingness to participate in frontline work is negatively correlated with burnout, stress, and anxiety

**Table 2:** 

Psychological condition	Factors associated with higher risk
Depression and anxiety	*Individual/interpersonal factors* (i) Female gender [14, 18, 29, 32, 34] (ii) Young age [18, 20, 29, 30, 34] (iii) Attention to negative information about the pandemic [13] (iv) Poor coping mechanisms [22] (v) Having experienced a previous pandemic [28] (vi) Lower level of education [22] (vii) Insufficient knowledge of COVID-19 [29] *Institutional factors* (i) Frontline work [12–14, 16, 18, 20, 28–30, 32, 35] (ii) Nonfrontline work [27] (iii) Loss of a patient due to COVID-19 [34] (iv) Loss of a close person from COVID-19 [20] (v) Higher workload [25] (vi) Less work experience [18] (vii) Lack of PPE or resources in the workplace [20, 26, 29] (viii) Administrative or paramedical work [20, 22] (ix) Working as a nurse [13, 16, 30, 32] *Community factors* (i) High media exposure [23] (ii) High-income countries [29] (iii) Lack of social support [33]

Posttraumatic stress disorder	*Individual/interpersonal factors* (i) Female gender [21, 23, 30, 32] (ii) Young age [30, 34] (iii) Passive coping strategies [21] (iv) Anxiety about being infection [24] *Institutional factors* (i) Frontline work [12, 21, 32] (ii) Loss of a patient due to COVID-19 [24, 34] (iii) Higher workload [25] (iv) Lower team cohesion [23] (v) Working as a nurse [21] *Community factors* (i) High media exposure [23] (ii) Social stigmatization [23, 33]

Burnout	*Individual/interpersonal factors* (i) Young age [30, 31] (ii) Higher alcohol intake [19] (iii) Unwillingness to participate in frontline work [33] (iv) Attention to negative information about the pandemic [13] *Institutional factors* (i) Frontline work [12, 13, 19, 30–32] (ii) Higher workload [30, 31] (iii) Less work experience [31] (iv) Lack of PPE or resources in the workplace [19, 26] (v) Conflicts in the workplace [31]

Insomnia	*Individual/interpersonal factors* (i) Female gender [14, 15, 32] (ii) Education level of high school or less [17] (iii) Attention to negative information about the pandemic [13] *Institutional factors* (i) Frontline work [13, 14, 32, 35] (ii) Nonphysician healthcare workers [15] (iii) Physicians [17] (iv) Higher workload [25] (v) Uncertainty about disease control [17]

## Data Availability

The data supporting this systematic review are from previously reported studies and datasets, which have been cited. Please refer to Table 1 in the manuscript for this data.

## Abbreviations

AIS: Athens Insomnia Scale
AUD: Alcohol use disorder
COVID-19: Coronavirus disease of 2019
DASS-21: Depression, Anxiety and Stress Scale 21
DSM-V: Diagnostic and Statistical Manual of Mental Disorders
ED: Emergency department
FL: Frontline
FLHCWs: Frontline healthcare workers
GAD: Generalized anxiety disorder
GAD-7: General anxiety disorder 7
HADS: Hospital Anxiety and Depression Scale
HCWs: Healthcare workers
ISI: Insomnia Severity Index
MERS: Middle East respiratory syndrome
MD: Major depression
OH: Alcohol
PHQ-9: Patient health questionnaire
PPE: Personal protective equipment
PRISMA: Preferred reporting items for systematic reviews and meta-analyses
PSQI: Pittsburgh Sleep Quality Index
PTS: Posttraumatic stress
PTSD: Posttraumatic stress disorder
PTSS: Posttraumatic stress symptoms
SARS-CoV-1: Severe acute respiratory syndrome coronavirus 1
SARS-CoV-2: Severe acute respiratory syndrome coronavirus 2
vs.: Versus
WHO: World Health Organization.
